# Unraveling *flp-11*/*flp-32* dichotomy in nematodes

**DOI:** 10.1016/j.ijpara.2016.05.010

**Published:** 2016-10

**Authors:** Louise E. Atkinson, Iain R. Miskelly, Christy L. Moffett, Ciaran J. McCoy, Aaron G. Maule, Nikki J. Marks, Angela Mousley

**Affiliations:** Microbes & Pathogen Biology, Institute for Global Food Security, School of Biological Sciences, Queen’s University Belfast, Belfast BT9 7BL, UK

**Keywords:** Neuropeptide, Neuropeptide signalling, FMRFamide-like peptide, Nematode, *flp-11*, *flp-32*, FLP-11, FLP-32

## Abstract

FMRFamide-like peptide (FLP) signalling systems are core to nematode neuromuscular function. Novel drug discovery efforts associated with nematode FLP/FLP receptor biology are advanced through the accumulation of basic biological data that can reveal subtle complexities within the neuropeptidergic system. This study reports the characterisation of FMRFamide-like peptide encoding gene-11 (*flp-11*) and FMRFamide-like peptide encoding gene-32 (*flp-32*), two distinct *flp* genes which encode the analogous peptide, AMRN(A/S)LVRFamide, in multiple nematode species – the only known example of this phenomenon within the FLPergic system of nematodes. Using bioinformatics, in situ hybridisation, immunocytochemistry and behavioural assays we show that: (i) *flp-11* and *-32* are distinct *flp* genes expressed individually or in tandem across multiple nematode species, where they encode a highly similar peptide; (ii) *flp-11* does not appear to be the most widely expressed *flp* in *Caenorhabditis elegans*; (iii) in species expressing both *flp-11* and *flp-32*, *flp-11* displays a conserved, restricted expression pattern across nematode clades and lifestyles; (iv) in species expressing both *flp-11* and *flp-32*, *flp-32* expression is more widespread and less conserved than *flp-11*; (v) in species expressing only *flp-11*, the *flp-11* expression profile is more similar to the *flp-32* profile observed in species expressing both; and (vi) FLP-11 peptides inhibit motor function in multiple nematode species. The biological significance and evolutionary origin of *flp-11* and *-32* peptide duplication remains unclear despite attempts to identify a common ancestor; this may become clearer as the availability of genomic data improves. This work provides insight into the complexity of the neuropeptidergic system in nematodes, and begins to examine how nematodes may compensate for structural neuronal simplicity. From a parasite control standpoint, this work underscores the importance of basic biological data, and has wider implications for the utility of *C. elegans* as a model for parasite neurobiology.

## Highlights

• *flp-11* expression pattern is restricted in *Caenorhabditis elegans.*
• *flp*-*11* expression is conserved and restricted across multiple nematodes.
• *flp*-*32* expression is more widespread and spatially species-specific.
• *flp-11* expression is more similar to the *flp-32* profile in species expressing only *flp-11.*
• FLP-11 peptides inhibit motor function in multiple nematode species.

## Graphical abstract

**Figure:**
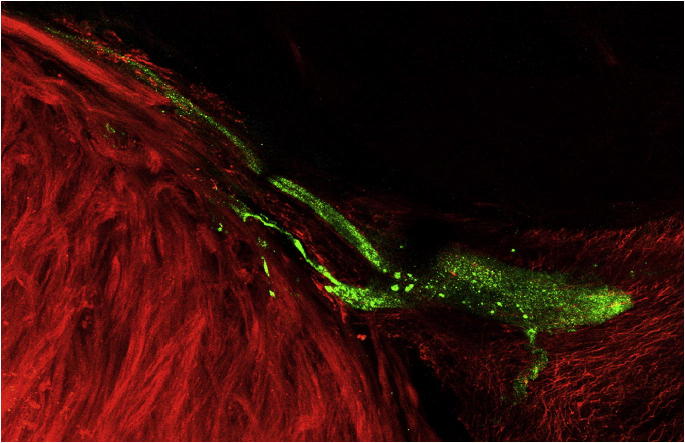

## 1 Introduction

FMRFamide-like peptides (FLPs) are the largest family of nematode neuropeptides. Current genomic data indicate the presence of 32 *flp* genes encoding >70 RFamide-like peptides (McCoy et al., 2014). Significantly, while *flp*-gene signatures are structurally conserved between nematode species irrespective of clade designation or lifestyle habit, *flp* gene complements appear to broadly map clade divisions, with some *flp* genes being conserved at the pan-phylum level (e.g. *flp-1* and *flp-14*), and others displaying restricted clade-specific (e.g. *flp-9*) or lifestyle-dependent (e.g. *flp-31*) conservation patterns (McCoy et al., 2014). Surprisingly, published data indicate that conservation in *flp*/FLP complement may not be synonymous with matching expression patterns. Indeed, published localisation studies for multiple *flp* genes in several nematode species reveal both positional similarities and differences in orthologous *flp* gene expression (Kimber et al., 2002, Yew et al., 2007, Jarecki et al., 2010, Jarecki et al., 2013, Marks and Maule, 2010, Sithigorngul et al., 2011), fuelling speculation that species-specific *flp*-expression patterns could be linked to behavioural traits that correspond with different habitats and/or life histories. However, while this may be the case, these localisation data were generated exclusively from single nematode species and directly compared with data generated for different species using alternative localisation techniques. This highlights the need for more rigorous comparative analyses of *flp* expression patterns across multiple nematode species using technique-matched approaches.

This study addresses these issues by mapping the expression of two *flp* genes (*flp-11* and *flp-32*) and their peptide products across multiple nematode species representing different clades and lifestyles through the use of in situ hybridisation (ISH) and immunocytochemistry (ICC). We selected *flp-11* which encodes three VRFamide peptides in *Caenorhabditis elegans* and *Ascaris suum*: AMRNALVRFG, (A)S(G)GMRNALVRFG and NGAPQPFVRFG (Husson et al., 2005, Yew et al., 2007) due to the reported disparity in spatial expression between *C. elegans* and *A. suum*. *flp-11* is reportedly the most widely expressed *flp*-gene in *C. elegans* (Kim and Li, 2004), whereas in *A. suum flp-11* expression is restricted to a single distinct cell (RIS) (Yew et al., 2007). This is in spite of the fact that the *C. elegans* and *A. suum* nervous systems have been described as scale models of each other (Nanda and Stretton, 2010). We also describe the occurrence and distinguishing features of an additional AMRN(A/S)LVRFamide-encoding gene (*flp-32*) in the phylum Nematoda and examine its expression across multiple species; distinct *flp* genes encoding almost identical FLPs (one amino acid difference) in nematodes is unusual.

The data presented here provide insight into the complexities of the neuropeptidergic system in nematodes, highlighting genuine similarities and differences in *flp* expression patterns across nematode species that are derived from technique-matched experiments, and appear to be gene-dependent. These data encourage the hypothesis that diversity in nematode behaviour and life history is partly supported by plasticity within their highly conserved neuropeptide systems. The findings underscore the importance of both breadth and depth in comparative analyses of nematode neuropeptide signalling systems and have wider implications for the utility of *C. elegans* as a model for parasite neurobiology and drug target discovery.

## 2 Materials and methods

### 2.1 Nematode preparation

*Caenorhabditis elegans* (strain N2) were obtained from the *Caenorhabditis* Genetics Centre, University of Minnesota, USA, and cultured on Nematode Growth Medium (NGM; 50 mM NaCl, 1.7% (w/v) agar, 0.25% (w/v) peptone, 1 mM CaCl_2_, 5 μg/ml of cholesterol, 1 mM MgSO_4_, 25 mM KH_2_PO_4_) agar plates, at 21 °C with *Escherichia coli* OP50 as a food source (Brenner, 1974). Mixed culture *C. elegans* were removed from NGM plates by washing in fresh M9 buffer (2.2 mM KH_2_PO_4_, 4.2 mM Na_2_HPO_4_, 8.5 mM NaCl, 1 mM 1 M MgSO_4_), and used immediately in experiments.

*Panagrellus redivivus* (strain MT8872) were obtained from the *Caenorhabditis* Genetics Centre, University of Minnesota, USA, and cultured on oatmeal slurry at 21 °C in complete darkness. Mixed stage *P. redivivus* were washed off the oatmeal slurry with M9 buffer and used immediately in experiments.

*Globodera pallida* (strain Pa2/3) were collected from potato plants of the Cara cultivar and maintained at the Agri-Food and Bioscience Institute (AFBI), Belfast, Northern Ireland. Pre-parasitic J2s were hatched from cysts in fresh potato root diffusate at 15 °C in complete darkness. Freshly hatched J2s were washed briefly in diethylpyrocarbonate (DEPC)-treated spring water and used immediately in experiments.

Ensheathed *Teladorsagia circumcincta* and *Haemonchus contortus* L3s were obtained from Moredun Research Institute, Penicuik, Scotland and stored at 4 °C. L3s from both species were brought to room temperature for at least 1 h, exsheathed (30 min wash in 0.2% sodium hypochlorite/PBS (5 M NaCl, 0.025 M NaH_2_PO_4_·2H_2_O, 0.075 M Na_2_HPO_4,_ pH 7.4) at room temperature), relaxed (30 min wash in 0.1 M gamma-aminobutyric acid (GABA) at room temperature), and used immediately in experiments.

Adult *A. suum* were obtained from a local abattoir (Karro Food Group, Northern Ireland) and transferred to the laboratory in mammal saline (0.9% NaCl). Worms were maintained in *Ascaris* Ringers Solution (ARS: 13.14 mM NaCl, 9.47 mM CaCl_2_, 7.83 mM MgCl_2_, 12.09 mM C_4_H_11_NO_3_/Tris, 99.96 mM NaC_2_H_3_O_2_,19.64 mM KCl, pH 7.8) at 37 °C prior to use in experiments.

### 2.2 Bioinformatics

A reciprocal Basic Local Alignment Search Tool (BLAST) based approach was employed to identify *flp-11* and *-32* sequelogs within publically available nematode genomic and transcriptomic datasets. This included species from clades 2, 4, 8, 9, 10 and 12 (Table 1). The BLAST analysis was conducted between July 2013 and May 2015; BLAST servers employed and databases queried are outlined in Table 1. Prepropeptide and protein sequences for *C. elegans flp-11* and *-32* were used as search strings in translated nucleotide (tBLASTn) and protein (BLASTp) BLAST analyses of available datasets. Predicted FLP-11 and -32 prepropeptide sequelogs were aligned using the Vector NTI Advance™11 AlignX® multiple sequence alignment tool (Lu and Moriyama, 2004), using default settings. Prepropeptide cleavage sites were identified using previously described prediction methods (McVeigh et al., 2005). Putative *flp-11* splice sites were identified by examination of the intron/exon boundaries (where adequate genomic information was available) associated with known *C. elegans* and *P. redivivus flp-11* splice variants. *flp-11* and *-32* putative N-terminal secretory signal peptides were identified using SignalP 4.1 (Petersen et al., 2011).

### 2.3 Molecular characterisation of *flp-11* and *flp-32* in nematodes

Full-length open reading frame (ORF) transcripts of *C. elegans flp-11* and *-32* (WBGene00001454 and WBGene00010982, respectively; http://www.wormbase.org) and *G. pallida flp-32* have previously been reported (Atkinson et al., 2013). The ORF sequences of *flp-11* and *-32* from *P. redivivus*, *G. pallida*, *T. circumcincta* and *H. contortus* were characterised using a range of PCR, degenerate PCR and Rapid Amplification of cDNA Ends (RACE) PCR techniques (Supplementary Fig. S1).

mRNA was extracted from *P. redivivus* (mixed culture), *G. pallida* J2s, *T. circumcincta* and *H. contortus* L3s using Dynabeads mRNA Direct™ kit (Life Technologies, USA) according to the manufacturer’s instructions. Separate populations of 5′ and 3′ RACE-ready cDNA were generated using the SMARTer™ RACE cDNA Amplification kit (Clontech, USA), as described in the manufacturer’s instructions.

*Panagrellus redivivus flp-11* (*Pr-flp-11*) and *T. circumcincta flp-11* (*Tc-flp-11*) were identified and characterised using degenerate primers (DGP; designed to the AMRNALVRFG and NGAPQPFVRFG encoding regions), the nematode splice leader primer (SL-1; *Tc-flp-11* only) and gene-specific primers (GSP) by PCR and RACE PCR. The ORFs of *G. pallida flp-11* (*Gp-flp-11*), *T. circumcincta flp-32* (*Tc-flp-32*) and *H. contortus flp-11* (*Hc-flp-11*) were characterised using GSPs designed against putatively assigned *Gp-flp-11*, and *Hc-flp-11* genome hits and a putative *Tc-flp-32* expressed sequence tag (EST; NCBI accession number CB037331) hits. The ORF of *H. contortus flp-32* (*Hc-flp-32*) was identified and characterised using GSPs designed to the *T. circumcincta flp-32* (*Tc-flp-32*) ORF (see Supplementary Table S1 for all primer sets).

DGP and GSP were used in 50 μl of PCRs to confirm the expression of the transcript of interest as follows: 5 μl of 10× PCR buffer (Life Technologies), 3 μl of MgCl_2_ (50 mM, Life Technologies), 1 μl of dNTP mix (10 mM, Promega, USA), 1 μl of each sense and antisense degenerate or GSP primer (20 μM), 1 μl of cDNA template, 0.3 μl of Platinum® *Taq* DNA Polymerase (5 U/μl, Life Technologies), ddH_2_O to 50 μl. Thermal cycling conditions were as follows: initial denaturation and ‘hot start’ at 94 °C for 2 min, followed by 40 cycles of 94 °C for 1 min, 55 °C for 1 min, and 72 °C for 1 min, with a final extension step of 72 °C for 7 min. Deviations from the above cycling conditions involved the alteration of annealing temperatures according to the thermal properties of specific degenerate primer combinations. The components of 50 μl of RACE PCRs were as follows: 5 μl of 10× PCR buffer (Life Technologies), 3 μl of MgCl_2_ (50 mM, Life Technologies), 1 μl of dNTP mix (10 mM, Promega), 2.5 μl of RACE-ready 5′ or 3′ cDNA template, 5 μl of 10× Universal Primer Mix (UPM), 1 μl of sense or antisense GSP (20 μM), 0.3 μl of Platinum® *Taq* DNA Polymerase (5 U/μl, Life Technologies), ddH_2_O to 50 μl. RACE PCRs were carried out using the thermal cycling conditions above with annealing temperatures of 60–65 °C.

All PCR products were viewed on a 1% agarose/Tris acetate EDTA (TAE) gel containing 0.0075% (v/v) ethidium bromide (10 mg/ml), and purified using a Charge Switch PCR Clean-up kit (Life Technologies). Products were cloned using the pCR®2.1 TOPO vector in One Shot® Chemically Competent TOP10 *E. coli* (Life Technologies), and sequence verified (at least three individual clones per PCR product; GATC Biotech; http://www.gatc-biotech.com). Return sequences were analysed using Vector NTI Advance Alignx (Life Technologies; Lu and Moriyama, 2004).

### 2.4 In situ hybridisation (ISH)

ISH probe templates (187–223 bp) were generated by PCR (cycling conditions and reaction mixtures as described in Section 2.3) using GSP (see Supplementary Table S1) designed to the *flp-11* and *flp-32* target transcripts in each nematode species, and a positive control (*Gp-flp-32*; Atkinson et al., 2013). Where appropriate, (*Ce-flp-11* and *Pr-flp-11*) ISH probes were designed to localise all known splice variants. ISH probe template PCR products were sequence verified as described in Section 2.3. Digoxigenin (DIG)-labelled single stranded DNA (ssDNA) probes with sense and antisense polarity were generated from PCR cleaned (ChargeSwitch® PCR Clean-Up Kit, Life Technologies) ISH probe templates by the LATE-PCR method (Hecker and Roux, 1996), in the following reaction: 5 μl of 10× PCR buffer (Life Technologies), 3 μl of MgCl_2_ (50 mM, Life Technologies), 2 μl of DIG dNTP mix (Roche, Switzerland), 1 μl each of sense and antisense primers (20 μM or 1 μM according to polarity of probe), 2 μl of corresponding ISH probe template, 0.3 μl of Platinum® *Taq* DNA Polymerase (5 U/μl, Life Technologies), and ddH_2_O to 50 μl. ISH was carried out using approximately 2000 *C. elegans* (mixed stage), *G. pallida* J2s, *P. redivivus* (mixed stage), *T. circumcincta* and *H. contortus* L3s according to methods previously described (Kimber et al., 2002, Atkinson et al., 2013), with the exception of a longer permeabilisation step (proteinase K, 50 min) for *T. circumcincta* and *H. contortus*. Hybridised probes were detected with substrate (5-bromo-4-chloro-3-indolyl phosphate/nitro blue tetrazolium tablet; BCIP/NBT, Sigma–Aldrich, USA) for up to 3 h at room temperature. Specimens were mounted on glass slides and photographed using a Leica DFC300FX camera and Leica FW4000 V 1.2 software with a Leica DMR light microscope.

### 2.5 Immunocytochemistry (ICC)

Polyclonal antisera were raised to two peptides encoded by *flp-11* and *-32* (anti-AMRNALVRFamide and anti-NGAPQPFVRFamide) in guinea pigs (Genosphere Biotechnologies, France), N-terminally coupled to keyhole limpet hemocyanin (KLH) and affinity purified. The anti-FMRFamide antiserum was raised as previously described (Fellowes et al., 1999). Approximately 1000 *C. elegans* (mixed stage), *G. pallida* J2s, *P. redivivus* (mixed stage), *T. circumcincta* and *H. contortus* L3s were immunostained using the indirect immunofluorescence technique (Coons et al., 1955), using methods previously described (Kimber et al., 2001). Primary antisera were used at 1/100 working dilution, and worms were counterstained for muscle with 200 ng/ml of phalloidin-tetramethylrhodamine isothiocyanate (TRITC). Nematodes were viewed on a Leica SP5 confocal scanning laser microscope. The omission of primary antiserum, replacement of primary antiserum with pre-immune serum from the donor species and pre-adsorption of the primary antiserum with ⩾250 ng of the appropriate antigen were included as negative controls. A positive control, pre-adsorption of anti-AMRNALVRFamide with ⩾250 ng of anti-NGAPQFPVRFamide and vice versa, was also carried out to ensure antibody selectivity. No immunostaining was observed in any of the negative control experiments. Immunostaining in the positive control experiments was consistent with that in the experimental samples.

### 2.6 FLP-11 behavioural assays

Behavioural assays to monitor the impact of FLP-11 peptides on locomotion and pharyngeal pumping in *P. redivivus* were based on methods previously described for *C. elegans* (Hart, 2006). Locomotion assays were carried out on NGM agar in the absence of a food source. Locomotion was assessed by counting body bends per min.

NGM agar (5 ml) was poured into 5 cm Petri dishes and allowed to solidify. Plates were stored at 4 °C until use and equilibrated to room temperature prior to experiments. FLP-11 peptides (AMRNALVRFamide, NGAPQPFVRFamide and AAGMRNALVRFamide; EZBiolab, Indianapolis, USA) were diluted in M9 buffer (final concentration 1 mM) and gently spread over the NGM agar. M9 buffer and serotonin (5-HT, Sigma–Aldrich; diluted to 100 mM in M9 buffer) were used as negative and positive controls, respectively. Prior to experiments worms were starved for 30 min on fresh OP50-free NGM agar plates at room temperature. Single adult *P. redivivus* and *C. elegans* were picked at random from a mixed population using a sterile platinum wire and placed onto on the experimental NGM plates containing M9, 5-HT or FLP-11 peptide, and were equilibrated for 30 min prior to recording. Each worm was recorded for a total of 3 min (three times for 1 min intervals; at least six worms per treatment were recorded for locomotion) using a Prior pro scanner platform, a Leica MZ 12.5 stereomicroscope and Unibrain Fire-i digital camera. Video clips were analysed by replaying each recording at half speed. When counting body bends per min, one body bend was classified as a maximum bend in the direction of the last bend counted (Horvitz et al., 1982). Data were analysed by one-way ANOVA and Tukey’s multiple comparisons post-test using GraphPad PRISM Version 6 package for Mac (GraphPad Software, Inc.). Data with probabilities of less than 5% (*P* < 0.05) were deemed statistically significant.

## 3 Results and discussion

The nematode species employed in this study (*C. elegans, P. redivivus, G. pallida, H. contortus, T. circumcincta*, and *A. suum*) represent different clades (8 (*A. suum*), 9 (*C. elegans, H. contortus, T. circumcincta*), 10 (*P. redivivus*), and 12 (*G. pallida*); Holterman et al., 2006), exhibit alternative lifestyles (free-living (*C. elegans* and *P. redivivus*), plant parasitic (*G. pallida*), and animal parasitic (*A. suum, H. contortus* and *T. circumcincta*)) and lifestages (mixed (*C. elegans* and *P. redivivus*), larval J2 (*G. pallida*), larval exsheathed L3 (*H. contortus* and *T. circumcincta*), and adult (*A. suum*)), and are species of agricultural (*G. pallida*), veterinary (*A. suum, H. contortus* and *T. circumcincta*), and economic significance. The range of life history traits and distinct lifestages represented by these species facilitates a broad, comparative examination of *flp-11* and *-32* characteristics in the phylum Nematoda.

### 3.1 FLPs display widespread neuronal distribution in nematodes

**Fig. 1:**
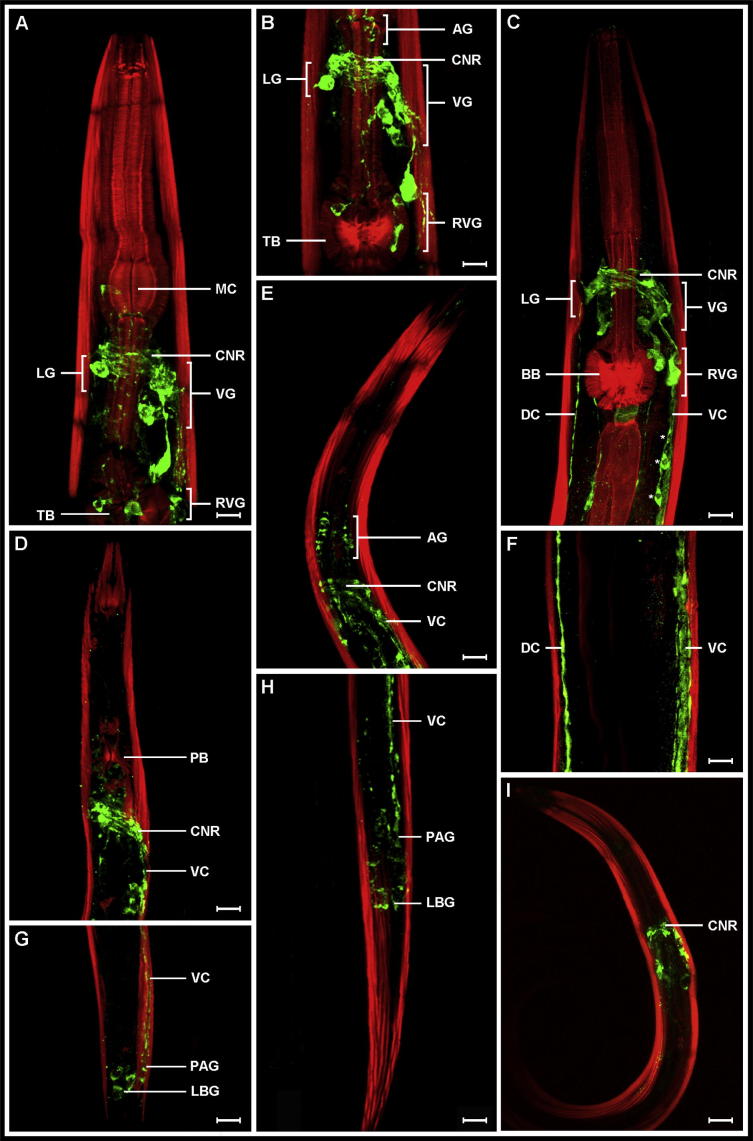
FMRFamide-like peptides (FLPs) are abundant in the nervous system of nematodes. Confocal microscopy images of FLP immunoreactivity (IR; green) in (A, B) *Caenorhabditis elegans*, (C, F) *Panagrellus redivivus*, (D, G) *Globodera pallida*, (E, H) *Teladorsagia circumcincta* and (I) *Haemonchus contortus*. Body wall muscle is counterstained in red. (A–E and I) Dense FLP-immunoreactivity (IR) in the circumpharyngeal nerve ring (CNR) of all nematode species examined. The CNR is positioned posterior to the metacorpus (MC) and anterior to the terminal bulb (TB) in *C. elegans* (A, B), anterior to the basal bulb (BB) in *P. redivivus* (C), and anterior to the pharyngeal bulb (PB) in *G. pallida* (D). Immunoreactive cell bodies in the major head ganglia (anterior ganglia (AG), lateral ganglia (LG), ventral ganglia (VG) and retrovesicular ganglion (RVG)) are shown (A–C), while FLP IR is also evident in the ventral (VC) and dorsal (DC) nerve cords which emanate from the CNR and run parallel to the body wall muscle (C–H). In addition, three FLP IR cell bodies (∗) can been seen in the VC of *P. redivivus* (C). FLP positive cell bodies are also shown in the region of the preanal (PAG) and lumbar (LBG) ganglia in the tail of *G. pallida* (G) and *T. circumcincta* (H). Scale bars: A = 7 μm, B = 6 μm, C = 10 μm, D = 40 μm, E = 20 μm, F = 8 μm, G = 35 μm, H = 36 μm, I = 40 μm.

FLPs are expressed extensively throughout the nervous systems of all of the nematode species examined. Anti-FMRFamide (Fellowes et al., 1999) immunoreactivity is particularly abundant in the numerous cell bodies and neuronal processes associated with the brain (Fig. 1A–E, I) and longitudinal nerve cords (Fig. 1C–H), a pattern consistent amongst species regardless of lifestyle, clade or life cycle stage. FLPergic cell bodies are located in the region of the circumpharyngeal nerve ring (CNR) within several of the main head ganglia including the anterior, lateral, ventral and retrovesicular ganglia (AG, LG, VG, RVG, respectively; Fig. 1A–E, I), and in the posterior region of the worm, in the preanal and lumbar ganglia (PAG, LBG; Fig. 1G, H). The VG and RVG comprise the largest collection of neurons in the *C. elegans* nervous system (Altun, Z.F., Hall, D.H. 2011. Nervous system, general description. In WormAtlas. http://dx.doi.org/10.3908/wormatlas.1.18) therefore it is not surprising that FLPs, which are thought to be responsible for various essential neuronal activities (see Day et al., 1994, Maule et al., 1995, Maule et al., 2002, Moffett et al., 2003, Kimber et al., 2007, Marks and Maule, 2010, Mousley et al., 2010), are localised in these regions. FLP immunostaining is also strong in the dorsal and ventral nerve cords (DC and VC), longitudinal nerve bundles containing neuronal processes extending from the main ganglia, connecting the CNR and PAG (Fig. 1C, F–H). In addition to the cell bodies contained in the major ganglia, a number of neuronal cell bodies also lie along the length of the VNC in a triplet arrangement as shown in Fig. 1C. Unlike the more specialised nerve tracks which have specific functions (e.g. amphid and phasmid nerves), the neuronal activities of the DC, VC and CNR have mixed functions (Altun, Z.F., Hall, D.H. 2011. Nervous system, general description. In WormAtlas. http://dx.doi.org/10.3908/wormatlas.1.18), consistent with the variety of roles attributed to FLPs.

FLP immunoreactivity appears to be generally more abundant in the free-living species *C. elegans* and *P. redivivus* than the plant parasitic nematode (PPN) *G. pallida* and the gastrointestinal nematodes *H. contortus* and *T. circumcincta* (Fig. 1). While this observation may be associated with reduced *flp* complement in these species (see McCoy et al., 2014), it may be a more accurate reflection of the lifestages employed here. For example *C. elegans* and *P. redivivus* mixed populations contain a high proportion of adult worms which perform a broad range of FLP modulated activities (locomotion, reproduction and feeding). In contrast the larval stages of *G. pallida*, *H. contortus* and *T. circumcincta* may have a reduced requirement for signalling molecules such as FLPs, or alternatively may require a more specific subset of FLPs involved in ‘priority’ lifestage-dependent functions (e.g. *G. pallida* J2s are likely to have a more focussed requirement for FLPs involved in sensory perception and locomotion to aid host plant directed orientation, invasion and migration, as opposed to reproduction and feeding). Nevertheless, these data demonstrate the abundance of FLPs in the nervous system of diverse nematodes, supporting the perceived importance of these peptides in neuromuscular function.

### 3.2 Two distinct *flp* genes, *flp-11* and *-32*, encode an analogous peptide in multiple nematode species

**Fig. 2:**
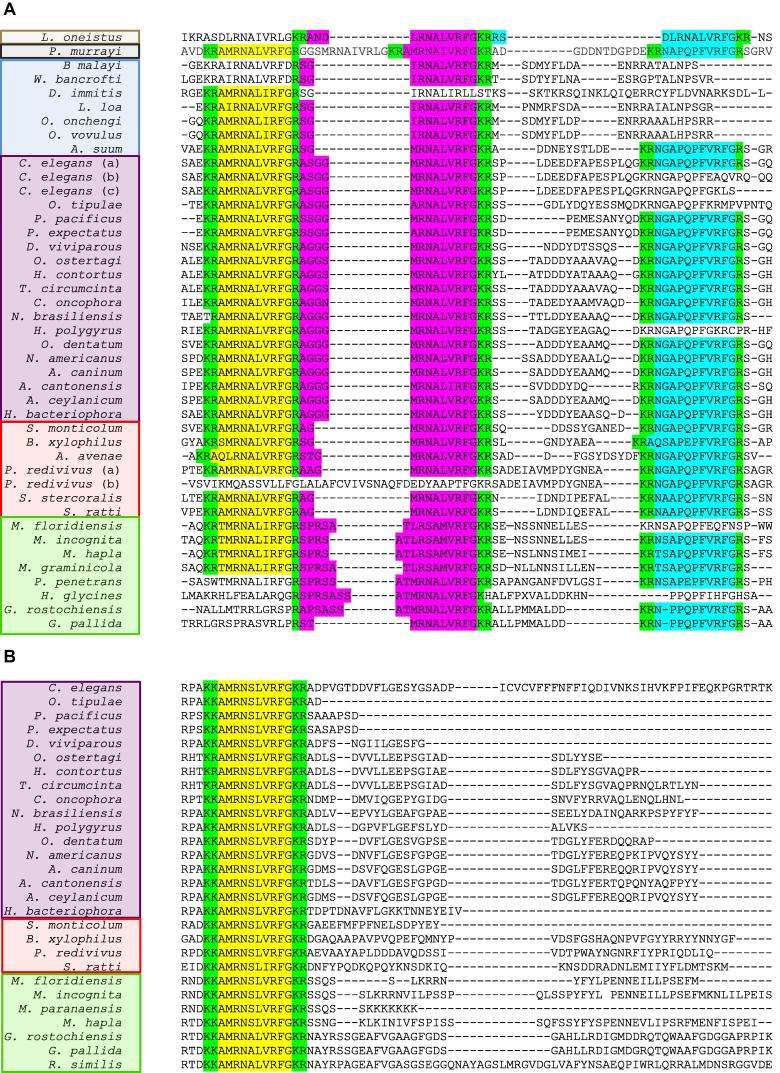
Alignment of partial sequence data from nematode species which express FMRFamide-like peptide encoding gene-11 (*flp-11*) and FMRFamide-like peptide encoding gene*-32* (*flp-32*). (A) Analysis of partial sequence data from nematode species which express *flp-11,* showing conservation of FMRFamide like peptide (FLP)-11-like peptides across 26 nematode species, demonstrating that up to three conserved peptides (highlighted in yellow, pink and blue) can be encoded. FLP-11-like peptide encoding transcripts display a highly conserved pattern of di- and mono-basic cleavage site residues (highlighted in green; with the exception of *Nippostrongylus brasiliensis* and *Heterodera glycines*), which characteristically incorporate a di-basic KR site preceding (A/T)(M/I)RNAL(V/I)RFG (highlighted in yellow), a mono-basic R residue prior to (A/S)(G/R/A)(G/S/T)(M/I)R(N/S)A(L/M)VRFG (highlighted in pink), and a di-basic KR and mono-basic R residue flanking (N/Q/T/Y)(G/S/A)AP(Q/E)PFVRFG. Clade 4 nematodes (*Laxus oneistus*) are boxed in brown; clade 6 nematodes (*Plectus murrayi*) are boxed in grey; clade 8 nematodes (*Brugia malayi, Wuchereria bancrofti, Dirofilaria imitis, Loa loa, Onchocerca ochengi, Onchocerca volvulus, Ascaris suum*) are boxed in blue; clade 9 nematodes (*Caenorhabditis elegans, Oscheius tipulae, Pristionchus pacificus, Pristionchus expectatus, Dictyocaulus viviparous, Ostertagia ostertagi, Haemonchus contortus, Teladorsagia circumcincta, Cooperia oncophora, N. brasiliensis, Heligmosomoides polygyrus, Oesophagostomum dentatum, Necator americanus, Ancylostoma caninum, Ancylostoma cantonensis, Ancylostoma ceylanicum, Heterorhabditis bacteriophora*) are boxed in purple; clade 10 nematodes (*Steinernema monticolum, Bursaphelenchus xylophilus, Aphelenchus avenae, Panagrellus redivivus, Strongyloides stercoralis, Strongyloides ratti*)*,* are boxed in red; clade 12 nematodes (*Meloidogyne floridensis, Meloidogyne incognita, Meloidogyne hapla, Meloidogyne graminicola, Pratylenchus penetrans, H. glycines, Globodera rostochiensis, Globodera pallida*) are boxed in green (Holterman et al., 2006). Species name suffix (a), (b) or (c) represents alternatively spliced gene isoforms. (B) Analysis of partial sequence data from nematode species which express *flp-32,* showing conservation of FLP-32-like peptides across 17 nematode species, demonstrating that only one conserved peptide (highlighted in yellow) is encoded. In contrast to cleavage site arrangement displayed by FLP-11-like peptide encoding transcripts, FLP-32-like peptide encoding transcripts characteristically encode two di-basic cleavage site residues (KK/R and KR; highlighted in green) flanking the single encoded peptide AMRN(A/S)L(V/I)RFG (highlighted in yellow). Clade 9 nematodes are boxed in purple; clade 10 nematodes are boxed in red; clade 12 nematodes (including *Meloidogyne paranaensis* and *Radopholus similis*) are boxed in green.

*flp-11* encodes at least one, but up to three peptides with a conserved L/FVRFG C-terminal motif, and was identified as one of the most highly represented nematode *flp* genes in a recent pan-phylum bioinformatics study (McCoy et al., 2014). We have expanded this trawl to evaluate the conservation of *flp-11* in all of the available genome and transcriptome datasets for free-living and parasitic nematodes. We show that *flp-11* is represented in 40 nematode species including clades 4, 6, 8, 9, 10 and 12; notably *flp-11* was not identified in any clade 2 nematode (Table 1; Fig. 2A). Of the three L/FVRFG peptides encoded by *flp-11*, AMRNALVRFG is the most highly conserved across species (Fig. 2A).

*Caenorhabditis elegans* possess three alternatively spliced forms of *flp-11*. *Ce-flp-11*a encodes AMRNALVRFG, ASGGMRNALVRFG and NGAPQPFVRFG, while *Ce-flp-11*b/c encode AMRNALVRFG and ASGGMRNALVRFG but not NGAPQPFVRFG (http://www.wormbase.org, Version WS247; Fig. 2A). The presence of alternatively spliced forms of *flp-11* appears to be a common theme across the nematode species. We have confirmed by PCR the presence of a different *flp-11* splice variant in the free-living nematode *P. redivivus*, which encodes NGAPQPFVRFG only (Fig. 2A). Further to this, BLAST analysis of the available nematode transcriptomic datasets reveals a number of species which appear to possess either a ‘*C. elegans*-like’ *flp-11* splice variant (NGAPQPFVRFG deficient), or, a ‘*P. redivivus*-like’ variant (NGAPQPFVRFG peptide only) as identified by the presence of conserved splice sites (data not shown).

In addition, we report the presence of a distinct *flp* gene (*flp-32*) that encodes a single peptide (AMRN(S/A)L(V/I)RFG) of striking similarity to the most common FLP-11 peptide AMRNAL(V/I)RFG. *flp-32* is present in 26 of the 40 species which possess *flp-11*, and in two species where *flp-11* has not been identified (Table 1). In addition *flp-32* is absent from all clade 2, 4, 6 and 8 species (Table 1). In the majority of nematode species that possess both genes, the peptides AMRN**A**LVRFG (FLP-11a) and AMRN**S**LVRFG (FLP-32) are most commonly encoded, such that there would be potential for co-expression of peptides with similar functions; this may indicate the importance of AMRNA/SLVRFamide to worm biology.

*flp-11* and *-32* can be distinguished by differences in: (i) peptide cleavage site arrangement and, (ii) genetic loci on the X chromosome of *C. elegans*. *flp-11* peptides are flanked by conserved cleavage sites where: peptide 1 (FLP-11a) has a dibasic cleavage site (KR) at the N-terminus and a monobasic cleavage site (R) at the C-terminus; peptide 2 (FLP-11b) shares the monobasic cleavage site at the C-terminus of peptide 1 (R) and has a C-terminal dibasic cleavage site (KR); peptide 3 (FLP-11c) has a dibasic cleavage site (KR) at the N-terminus and a C-terminal monobasic cleavage site (R) (Fig. 2A). The arrangement of *flp-11* peptide cleavage sites is almost completely conserved (Fig. 2A). In contrast, *flp-32* displays an alternative cleavage site arrangement whereby AMRNS/ALVRFG is flanked by C-terminal dibasic residues KK, and an N-terminal dibasic cleavage site, KR (Fig. 2B). This pattern is conserved in all *flp-32* genes with the exception of *H. contortus* and *T. circumcincta,* which instead have a KR cleavage site at the C-terminus.

While many nematode FLPs possess characteristically similar C-terminal motifs (see McCoy et al., 2014), FLP-11 and -32 are the only example of almost complete conservation of two peptides encoded by distinct *flp* genes in nematodes. The functional and evolutionary significance of this is not yet understood. Examination of *C. elegans flp* gene loci has confirmed unequivocally that *flp-11* and *-32* are distinct genes that map to alternative loci on the X chromosome of *C. elegans* (*flp-11*, X:4677342..4678129; *flp-32* X:15433787..15434970; http://www.wormbase.org, Version WS247), and are not simply the products of alternative splicing. Unfortunately the genomic positions of *flp-11* and *-32* appear to be poorly conserved in parasitic nematodes, therefore the identification of a conserved *flp-11* and *-32* genomic environment was not possible.

Given the sequence similarity displayed by *flp-11* and *-32* peptide products it is possible that these genes represent paralogous sequences that have evolved via a gene duplication event. *flp-11* and *-32* sequelogs were not identified within any clade 2 nematode species with a published genome (Table 1; Mitreva et al., 2011, Schiffer et al., 2013, Foth et al., 2014, Jex et al., 2014), suggesting that the last common ancestor of all nematodes may not have possessed either gene. Further, the clade level conservation of *flp-32* sequelogs (clades 9, 10 and 12) is more restricted than *flp-11* (clades 4, 6, 8, 9, 10 and 12; Table 1), suggesting that *flp-11* could represent the ancestral gene sequence. Together these observations imply that a gene duplication event may have resulted in the evolution of *flp-32* within the lineage of the last common ancestor of clades 9, 10 and 12 (Table 1). Alternatively, it is possible that a more complicated evolutionary history exists involving several independent losses of either or both genes within separate nematode lineages. In this scenario either gene could represent the ancestral sequence.

In an effort to investigate these hypotheses we examined the available genomic and transcriptomic data from the more basal nematode species (clades 1–7; Holterman et al., 2006). Unfortunately this analysis was restricted by the lack of representative draft genomes for the majority of these basal clades (excluding clade 2). Despite this, we did identify a *flp-11*/*32* sequelog within the transcriptomic data derived from the free-living marine nematode, *Laxus oneistus* (clade 4). This sequence displays characteristics similar to both *flp-11* and *-32* genes. This makes its unequivocal designation as either gene impossible and indicates that it could be representative of an ancestral *flp-11*/*32* gene (Fig. 2A). Alternatively, it may represent a divergent *flp* sequence. Unfortunately, the nematode genomic data currently available are not sufficient to determine conclusively the origin of *flp-11* and *-32*, however as these datasets progress the evolutionary history of *flp-11* and *-32* may become clearer.

### 3.3 *flp-11* does not appear to be the most widely expressed *flp* in *C. elegans*

**Fig. 3:**
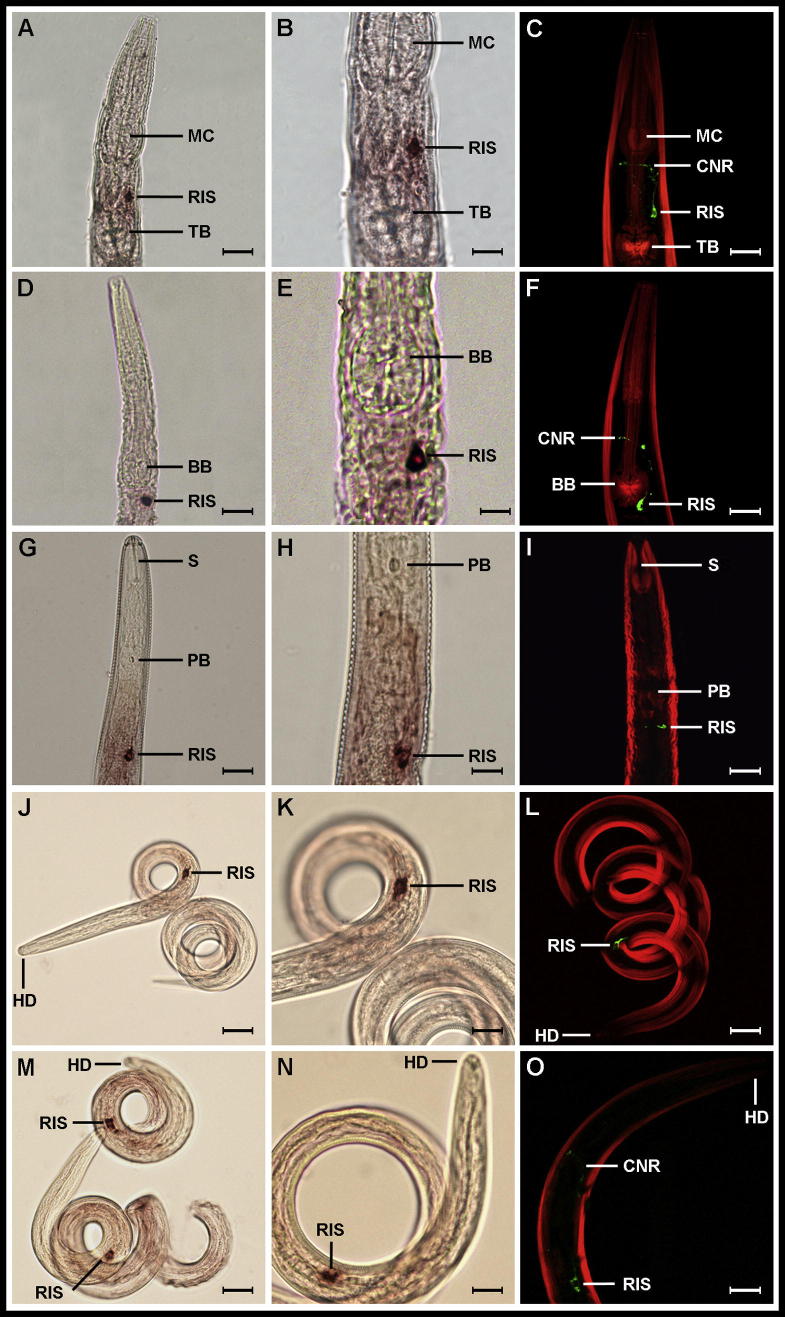
FMRFamide-like peptide encoding gene-11 (*flp-11*)/FMRFamide-like peptide (FLP)-11 expression is restricted to a single RIS-like (RIS) cell in nematodes. Light microscopy in situ hybridisation images show *flp-11* gene expression in a single RIS cell body positioned in the right side of the ventral ganglion in (A, B) *Caenorhabditis elegans*, (D, E) *Panagrellus redivivus*, (G, H) *Globodera pallida*, (J, K) *Teladorsagia circumcincta* and (M, N) *Haemonchus contortus*. RIS is positioned between the metacorpus (MC) and terminal bulb (TB) in *C. elegans* (A–C), posterior to the basal bulb in *P. redivivus* (D–F)*,* and posterior to the pharyngeal bulb (PB) and stylet (S) in *G. pallida* (G, H). In *T. circumcincta* (J, K) and *H. contortus* (M, N) RIS is shown in the anterior third of the nematode in proximity to the head (HD). (C, F, I, L and O) Confocal microscopy of FLP-11 (anti-NGAPQFPVRFamide) immunoreactivity (green) in *C. elegans* (C), *P. redivivus* (F), *G. pallida* (I), *T. circumcincta* (L) and *H. contortus* (O) in a single RIS-like cell (RIS). An anterior neuronal projection running into the circumpharyngeal nerve ring (CNR) is also evident in *C. elegans* (C), *P. redivivus* (F) and *H. contortus* (O)*.* Body wall muscle is counterstained red. Scale bars: A = 20 μm, B = 10 μm, C = 20 μm, D = 25 μm, E = 6 μm, F = 25 μm, G = 15 μm, H = 7 μm, I = 15 μm, J = 20 μm, K = 8 μm, L = 15 μm, M = 20 μm, N = 10 μm, O = 10 μm.

*Caenorhabditis elegans* GFP reporter gene fusion studies revealed the expression pattern of *flp-11* (*Ce-flp-11*) in ⩾44 neurons throughout the entire nematode body (Kim and Li, 2004). Subsequently, *flp-11* was regarded as the most widely expressed *flp* gene in nematodes. In this study, we show a highly restricted *C. elegans flp-11* expression (ISH (*Ce-flp-11*)) and localisation (ICC (*Ce*-FLP-11) using a FLP-11 specific anti-NGAPQPFVRFamide antiserum) pattern is limited to a single anterior neuronal cell body (Fig. 3A–C).

The *Ce*-*flp-11* positive cell reported here (positioned anterior to the terminal bulb in the region of the ventral ganglia; see Fig. 3A–C) is likely to be the single cell body associated with the RIS interneuron. RIS is one of only four unpaired neurons in the *C. elegans* VG and is characteristically positioned between the terminal and metacarpal bulbs, with a single moderately large neuronal process running anteriorly into the right side of the CNR (Fig. 3A–C) (Altun, Z.F., Hall, D.H. 2005. Cell identification in *C. elegans*. In *WormAtlas*. http://www.wormatlas.org/cellID.html). The restricted *Ce-flp-11* expression profile reported here aligns closely with the localisation (ICC) and expression (ISH) data for *flp-11* and its peptide products (AMRNALVRFamide and NGAPQPFVRFamide) in *A. suum* (Yew et al., 2007). In addition, unpublished data presented at the 2008 *C. elegans* Neuronal Development, Synaptic Function, and Behaviour Meeting (referenced on Wormbase) details *flp-11*::GFP reporter gene localisation, in a single cell near the posterior bulb of the *C. elegans* pharynx (Bhatla, N., Ringstad, N., Horvitz, B. 2008. Reduction of Movement by *flp-11*, which Encodes FMRFamide-related Peptides. *C. elegans* Neuronal Development, Synaptic Function, and Behaviour Meeting, University of Wisconsin, Madison, USA, June 29th–July 2nd, http://www.wormbase.org/db/get?name=WBPaper00032809;class=Paper) and, more recently, *flp-11* has been shown to be expressed in the RIS neuron using the *flp-11*::mKate2 reporter construct (Turek et al., 2016). Interestingly, *flp-11*::GFP expression was not identified in RIS in the *C. elegans* GFP reporter gene study by Kim and Li (2004); this discrepancy could be attributed to the known limitations of transcriptional reporters (Li and Kim, 2014), which may not accurately reflect target expression due to the potential for the exclusion of regulatory information and the influence of postranscriptional events and/or miRNAs on endogenous expression (Boulin et al., 2006). The data presented here confirm the RIS expression observed by Bhatla et al. (2008, *C. elegans* Neuronal Development, Synaptic Function, and Behaviour meeting, cited earlier) and Turek et al. (2016) using two different localisation methods (ISH and ICC). Collectively, these data lead us to propose that *flp-11* is not the most widely expressed *flp* in *C. elegans*, but instead may represent one of the more restricted *flp* genes.

### 3.4 *flp-11* displays a conserved, restricted expression pattern across nematode clades and lifestyles in species expressing both *flp-11* and *flp-32*

In the nematode species expressing both *flp-11* and *flp-32* that were examined in this study (*C. elegans*, *P. redivivus*, *G. pallida*, *H. contortus, T. circumcincta*), *flp-11*/FLP-11 expression and localisation patterns are strikingly conserved. Without exception, *flp-11*/FLP-11 in these species was expressed in a single cell body and anteriorly directed neuronal process running into the CNR (Fig. 3). While unequivocal assignment of neuronal cell bodies is difficult in nematodes lacking a neuronal map, the highly conserved and restricted expression pattern shown here (see Fig. 3), coupled with previous reports of *flp-11* expression in *A. suum* (Yew et al., 2007), and *C. elegans* (Bhatla et al., 2008, *C. elegans* Neuronal Development, Synaptic Function, and Behaviour meeting, cited earlier), supports the conclusion that this is likely to be the single cell body associated with the RIS interneuron. The *flp-11* ISH expression patterns described here are matched by ICC data (anti-NGAPQFPVRFamide directed antiserum; Fig. 3C, F, I, L, O). It should be noted that where sex differentiation was undertaken (e.g. *P. redivivus*), there were no observable sex-related differences in *flp-11*/FLP-11 expression.

Although we know little about the neuronal morphology of most of the nematodes examined in this study, *C. elegans* and *A. suum* are known to display homology with respect to cellular morphology and synaptic connections (Nanda and Stretton, 2010). This supports the extrapolation of the RIS cellular designation to the nematodes examined here, and furthermore, may indicate conservation of neuronal morphology, and potentially neuronal signalling processes across the nematode phylum.

While we show that the inter-species expression patterns of *flp-11*/FLP-11 appear to be consistent in distinct nematode species (Fig. 3A–O), the available literature on *flp*/FLP expression and localisation provides complex and incomplete data for other *flps*/FLPs, identifying both similarities and differences in expression (Marks and Maule, 2010). This highlights the possibility that *flp*/FLP expression/localisation may in some instances correlate with nematode inter-species diversity, or alternatively, as in the case of *flp-11*/FLP-11, may be conserved regardless of species-specific characteristics. One caveat is that published *flp*/FLP expression studies have employed a range of contrasting localisation techniques (GFP-reporter data, ISH, ICC) in a limited number of species; this bolsters the need for a multi-species, technique matched comparative approach to *flp*/FLP expression analyses. Indeed prior to the present study, which has adopted a technique matched approach, the available *flp-11* expression data were contradictory (Kim and Li, 2004, Yew et al., 2007; Bhatla et al., 2008, *C. elegans* Neuronal Development, Synaptic Function, and Behaviour meeting, cited earlier).

### 3.5 *flp-32* expression is more widespread than *flp-11* in those species expressing both *flp-11* and *flp-32* and appears to be species-specific

**Fig. 4:**
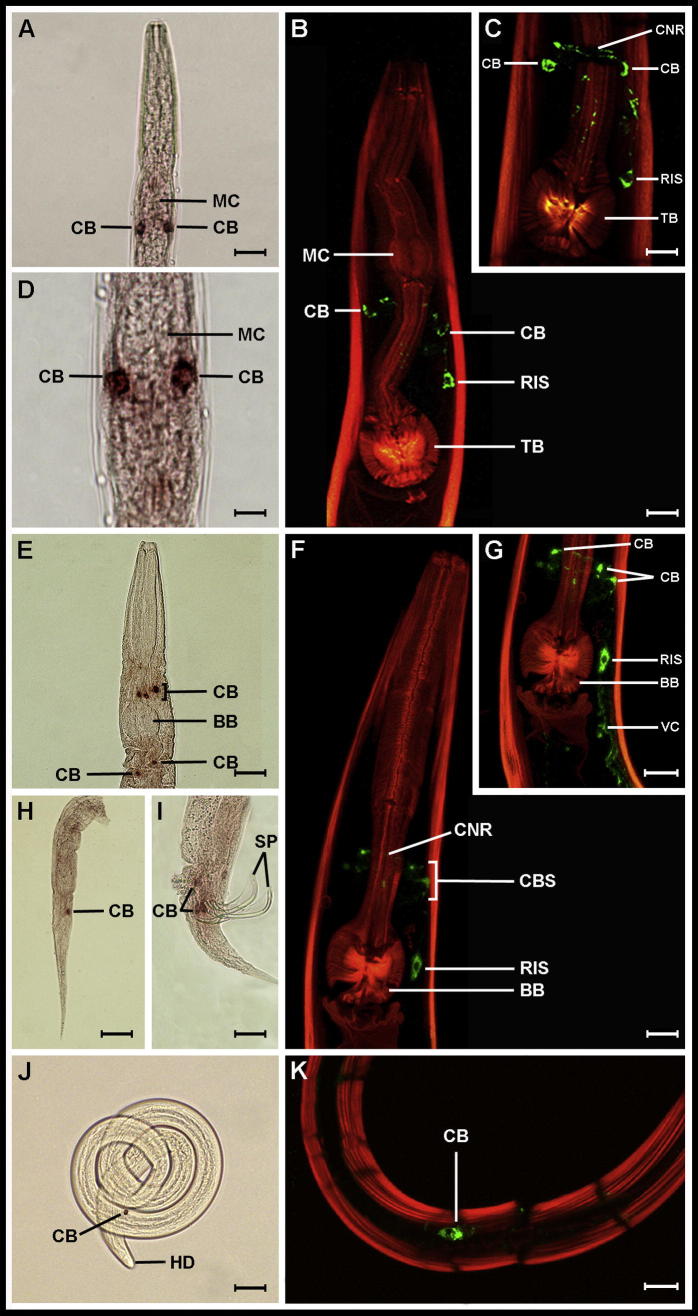
FMRFamide-like peptide encoding gene-32 (*flp-32*)/FMRFamide-like peptide (FLP)-32 expression is more extensive and less conserved than *flp-11*/FLP-11. Light microscopy in situ hybridisation images show *flp-32* gene expression in multiple cell bodies (CB) in (A, D) *Caenorhabditis elegans*, (E, H, I) *Panagrellus redivivus* and (J) *H. contortus*. In *C. elegans* two paired *flp-32* positive CBs are visible posterior to the metacorpus (MC). In *P. redivivus* three *flp-32* positive CBs are located anterior to the basal bulb (BB; E), while one CB is visible in the tail of a female nematode (H), and at least three CBs are in close proximity to the spicules in males (I). In *H. contortus* one CB is positioned in the posterior third of the nematode body (J). (B, C, F, G and K) Confocal microscopy of FLP-32 (anti-AMRNALVRFamide) immunoreactivity (IR; green) in *C. elegans* (B and C), *P. redivivus* (F and G) and *H. contortus* (K) in a single RIS-like cell (RIS) and in multiple additional cell bodies (CBs). Anti-AMRNALVRFamide antiserum is cross-reactive for both FLP-11 and FLP-32 explaining the presence of the RIS-like cell in the confocal microscopy images. In *C. elegans* (B, C), in addition to RIS, two FLP-32 IR CBs are visible in close association with the circumpharyngeal nerve ring (CNR) positioned between the MC and the terminal bulb (TB). In *P. redivivus* (F, G) FLP-32 positive CBs are evident in the region of the CNR, anterior to the BB and RIS; in addition the ventral nerve cord (VC) is FLP-32 immunoreactive (G). In *H. contortus* one FLP-32 IR cell is shown (K). Body wall muscle is counterstained red. Scale bars: A = 15 μm, B = 8 μm, C = 5 μm, D = 6 μm, E = 25 μm, F = 7 μm, G = 30 μm, H = 25 μm, I = 6 μm, J = 20 μm, K = 6 μm.

Unlike *flp-11*, there are no GFP reporter construct data available for *flp-32* in *C. elegans*. *flp-32* expression has been reported in the PPN *G. pallida*, where it was widely expressed in the brain, ventral nerve cord and tail (Atkinson et al., 2013). This is dramatically different to the highly restricted *flp-11* expression pattern (Fig. 3). This trend is conserved across nematodes where the expression (ISH) and localisation (ICC) of *flp-32*/FLP-32 is generally more widespread than *flp-11*/FLP-11 (Fig. 4A–K). In addition, while *flp-11*/FLP-11 expression is completely conserved in those species with both *flp-11* and *flp-32*, the expression of *flp-32*/FLP-32 is highly variable between species (Fig. 4A–K). *Caenorhabditis elegans* and *P. redivivus* express *flp-32* in multiple cells located in the anterior head region; in *C. elegans* these consist of a set of large paired cells in the region of the CNR between the metacorpus and terminal bulb (Fig. 4A, D), while in *P. redivivus* a total of five cells are located above (three cells) and below (two paired cells) the basal bulb (Fig. 4E). In addition, female *P. redivivus* possess one *flp-32* positive cell in the tail, while male worms display multiple *flp-32* cells closely associated with the spicules (Fig. 4H, I). In contrast, *H. contortus flp-32* is expressed in a single cell positioned in the posterior half of the nematode body (Fig. 4J).

The *flp-32* ISH expression patterns described above are matched by ICC data generated using an anti-AMRNALVRFamide antiserum (Fig. 4B, C, F, G, K). Anti-AMRNALVRFamide facilitates the localisation of both FLP-11 and -32 peptides (AMRNA/SLVRFG) whereas anti-NGAPQFPVRFamide (used in FLP-11 localisation, Fig. 3) is FLP-11 specific. Consequently, the FLP-32 ICC images in Fig. 4 are positive for both the single ‘RIS-like’ cell (identified in the FLP-11 ICC; Fig. 3C, F, I, L, O), and additional cells which are FLP-32 positive but were not anti-FLP-11 immunoreactive.

These data indicate that *flp-32* is not co-expressed in the same cell (RIS) as *flp-11* but instead has a completely contrasting expression pattern in a range of *flp-11* deficient cells. This observation is consistent with published data showing an array of *flp-32*/FLP-32 positive cells in *G. pallida* that did not include a ‘RIS-like’ cell (Atkinson et al., 2013). Experimental ICC controls included anti-NGAPQFPVRFamide pre-absorbtion with AMRNALVRFamide and vice versa to ensure antibody selectivity.

### 3.6 In *A. suum*, which expresses only *flp-11, flp-11* expression is more widespread than those species expressing both *flp-11* and *flp-32*

**Fig. 5:**
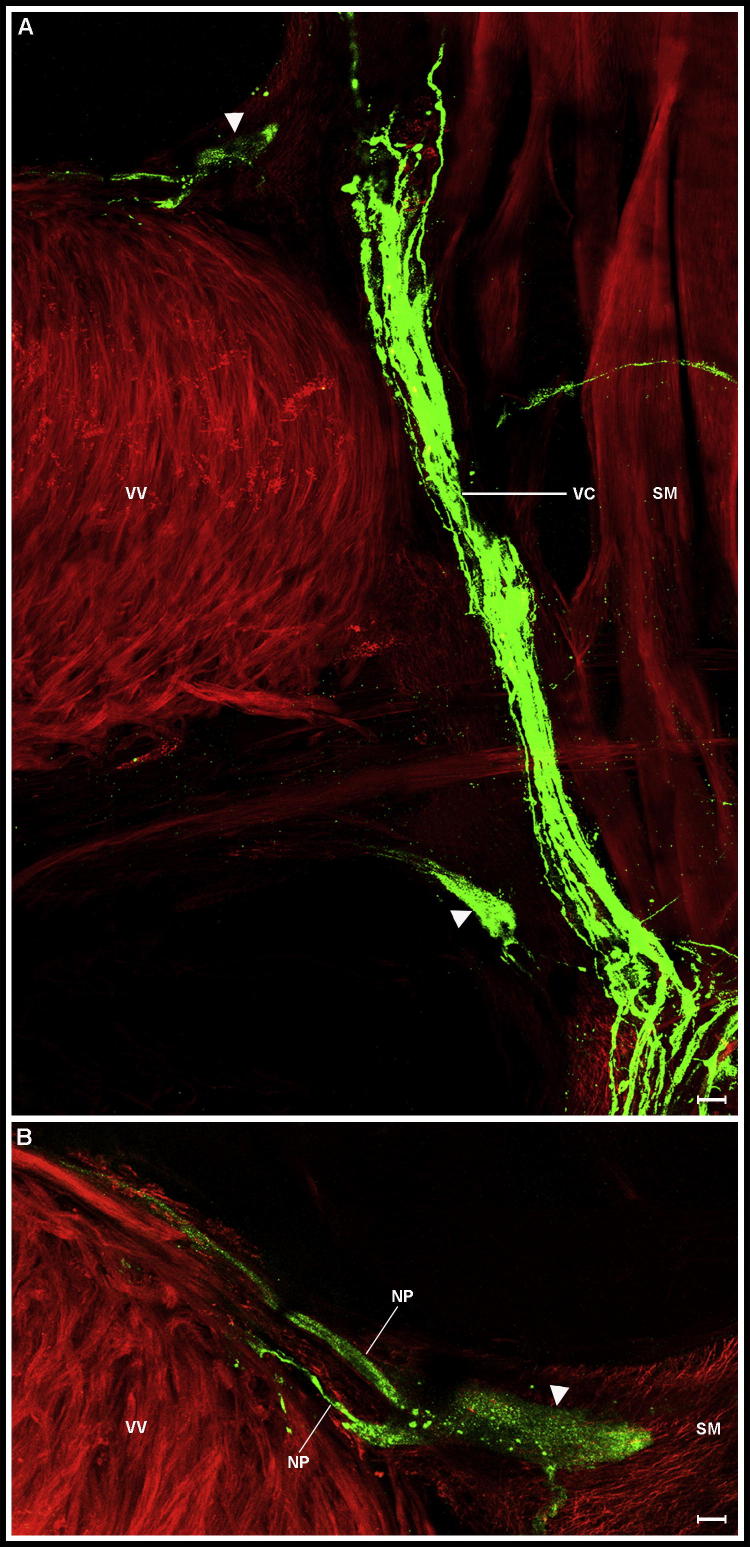
FMRFamide-like peptide (FLP)*-*11 is localised to two neuronal cell bodies associated with reproductive muscle and the ventral nerve cord (VC) in *Ascaris suum*. Immunocytochemistry images showing FLP-11 localisation in the region of the *A. suum* reproductive apparatus. (A) Two FLP-11 (anti-NGAPQFPVRFamide) immunoreactive (IR; green) cell bodies (arrows) located at the base of the ovijector in the region of the vagina vera muscle (VV; counterstained red), in close proximity to the FLP-11 positive VC which runs parallel to the VV/body wall junction. (B) Two FLP-11-IR neuronal processes (NP) extending from the cell body (arrow) and innervating the VV muscle. Somatic muscle (SM) is also counterstained red (A, B). Scale bars: A = 30 μm, B = 14 μm.

Some nematode species appear to possess only *flp-11* in that they are *flp-32* deficient. This is particularly noticeable within the Clade 8 nematodes where all seven representatives examined in this study conform to this trend. *Ascaris suum* is the only clade 8 nematode for which *flp-11* expression data have been mapped (Yew et al., 2007); *flp-11* was previously reported to be expressed in a single RIS-like cell in the ventral ganglion, conforming to the expression pattern observed for *flp-11* in all of the other nematode species examined in this study. Interestingly, FLP-11 peptides have been shown to have functional activity within the reproductive (ovijector) tissue of *A. suum* (Moffett et al., 2003), prompting the examination of *flp-11* expression in the ovijector region. The *A. suum* ovijector has previously been shown to be FLP-immunoreactive (anti-FMRFamide; Fellowes et al., 1999), where two FLP-positive cells, likened to *C. elegans* VC4 and VC5 (White et al., 1986), are located at the base of the ovijector and are connected to a FLPergic nerve plexus on the reproductive muscle. In this study, we localised FLP-11 (anti-NGAPQFPVRFamide) to these cells (Fig. 5). This indicates that *flp-11*/FLP-11 has a more widespread expression pattern in *A. suum* than all other nematodes examined in this study. This could be linked to the absence of *flp-32* in *A. suum*, as the other nematode species all possess both *flp-11* and *flp-32*. It is tempting to hypothesise that in all of those nematode species where *flp-32* is absent, *flp-11* expression is widespread, consistent with functional compensation.

### 3.7 FLP-11-like peptides inhibit motor function in multiple nematode species

**Fig. 6:**
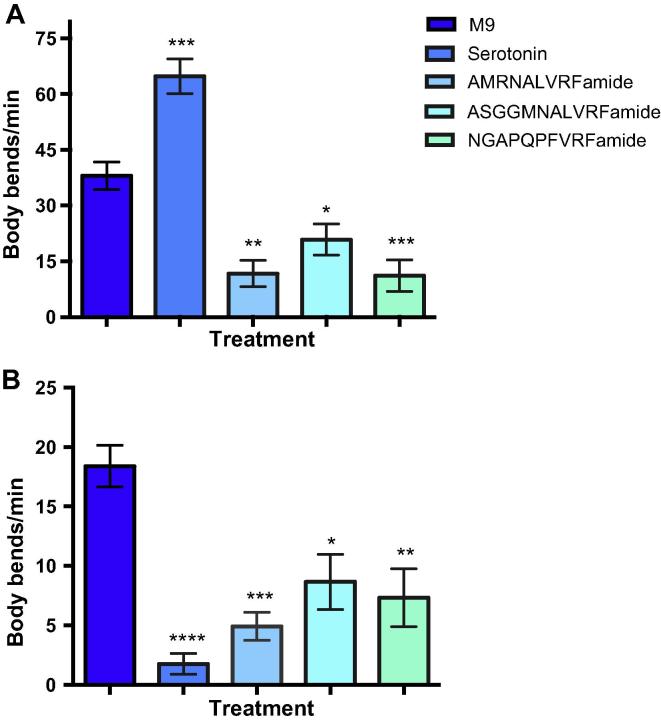
FMRFamide-like peptide (FLP)-11-like peptides inhibit locomotion in *Panagrellus redivivus* and *Caenorhabditis elegans*. FLP-11 peptides (AMRNALVRFamide, ASGGMNALVRFamide and NGAPQPFVRFamide) significantly inhibit locomotion in *P. redivivus* and *C. elegans.* (A) In *P. redivivus,* AMRNALVRFamide and NGAPQPFVRFamide induced the greatest inhibition of locomotion as assessed by a reduction in body bends/min (AMRNALVRFamide, 11.73 ± 3.55 body bends/min; ASGGMNALVRFamide 20.89 ± 4.15 body bends/min; NGAPQPFVRFamide, 11.17 ± 4.22 body bends/min) compared with control (M9 negative control, 38.06 ± 3.75 body bends/min; *P* < 0.01). Serotonin was used as a positive control (64.82 ± 4.66 body bends/min). (B) In *C. elegans,* AMRNALVRFamide induced the greatest inhibition of locomotion as assessed by a reduction in body bends/min (AMRNALVRFamide, 4.93 ± 1.17 body bends/min; ASGGMNALVRFamide 8.67 ± 2.32 body bends/min; NGAPQPFVRFamide, 7.33 ± 2.44 body bends/min) compared with control (M9 negative control, 18.40 ± 1.74 body bends/min; *P* < 0.01). Serotonin was used as a positive control (1.78 ± 0.88 body bends/min). ^*^P ⩽ 0.05; ^**^P ⩽ 0.01; ^***^P ⩽ 0.001.

Over half of the nematode species that possess *flp-11* also possess *flp-32* (see Table 1) such that many species have two genes expressing highly similar peptides. Whilst relatively little is known about the biological function of *flp-11* and *-32*, available evidence suggests that FLP-11 and -32 peptides play an inhibitory role. For example in *C. elegans, flp-11* overexpression mutants display a reduced movement phenotype (Bhatla et al., 2008, *C. elegans* Neuronal Development, Synaptic Function, and Behaviour meeting, cited earlier) and FLP-11 has been linked to sleep-like behaviour (Turek et al., 2016), while *flp-32* silencing experiments in *G. pallida* (Atkinson et al., 2013) show that *flp-32* inhibits locomotion in PPNs. However, there are subtle differences in FLP-11 peptide function/potency, which appear to be species- and/or tissue-specific. In *A. suum,* FLP-11 peptides (FLP-11a, b, c) have been shown to be inhibitory in whole worm, body wall muscle, motor neuron and pharyngeal activity assays, with FLP-11c (NGAPQPFVRFamide) producing the most suppressive and persistent effects (Yew et al., 2007, Reinitz et al., 2011). In keeping with this, when tested on ovijector tissue, NGAPQPFVRFamide also induced an inhibitory effect consisting of ovijector shortening and a cessation of contractile activity (response-type 1 (RT-1)), however this was less potent than the dominant and contrasting excitatory effect of the co-encoded FLP-11a peptide (AMRNALVRFamide) which decreased tissue length and contractile amplitude but increased contraction frequency (RT-5) (Moffett et al., 2003). This tissue-specific shift in FLP-11 peptide action and potency highlights the potential for target-dependent peptide effects which could be mediated by differences in the site of peptide release and/or downstream signalling effectors. These observations suggest subtle functional differences between peptides encoded on the same gene (*flp-11*) in *A. suum*. Since *A. suum* lacks *flp-32* we examined the effects of FLP-11 peptides on locomotion in *C. elegans* and *P. redivivus*, free-living nematodes that express both *flp-11* and *-32*. All FLP-11 peptides (AMRNALVRFamide, ASGGMNALVRFamide and NGAPQPFVRFamide; 1 mM) significantly inhibited locomotion in *P. redivivus* with NGAPQPFVRFamide and AMRNALVRFamide displaying the most significant inhibitory effects compared with the negative control (M9 buffer) (Fig. 6A). In *C. elegans* FLP-11 peptides were also inhibitory (Fig. 6B).

These data align with previously published experiments that indicate an inhibitory role for FLP-11 peptides in nematodes (Moffett et al., 2003, Yew et al., 2007, Reinitz et al., 2011, Turek et al., 2016); however, more detailed examinations of peptide function reveal subtle differences in activity and potency between peptides encoded on the same gene (within and between species). Unfortunately the complicated arrangement of *flp-11* and *-32* genes in nematodes (i.e. *A. suum* possesses *flp-11* but not *flp-32*; *G. pallida* possesses both genes but *flp-11* does not encode AMRN**A**LVRFG while *flp-32* does; *P. redivivus* encodes both genes but expresses multiple splice variants of *flp-11*) makes the comparison of available functional data challenging. Technique matched experiments are required to unravel the functional biology of *flp-11* and *-32* and their respective peptide products in a broad a range of nematode species.

In conclusion, this study describes the pan-phylum comparative analyses of *flp-11* and *-32* neurobiology in nematode species representing multiple clades and divergent life histories. We provide data to support the complexity of the neuropeptidergic system in nematodes by highlighting both similarities and differences in *flp* gene expression between divergent nematodes species, and genes that encode highly similar peptides. These data may begin to explain how nematodes compensate for structural simplicity and highlight the potential for plasticity in the neuropeptidome contributing to their behavioural sophistication.

This study is also significant with respect to the utility of *C. elegans* as a model nematode for parasite neurobiology, and from a parasite control perspective. The candidature of the neuropeptidergic system as a repository for novel putative drug targets has been previously discussed (see McVeigh et al., 2011), with the importance of FLPs to normal parasite behaviour underpinning the appeal of the FLPergic system as a therapeutic target resource. However, the expectation that a novel target will have broad-spectrum activity cannot be assumed or predicted from a model nematode (*C. elegans*) where there are parasite-specific differences in target expression patterns and/or function. Therefore, comparative analyses in parasite species (including key pathogens) representing multiple clades and lifestyles are critical to target validation processes, even within signalling systems which are perceived to be highly conserved between species. Indeed, unexpected neuronal variation is evident amongst diverse nematode species where a number of examples diverge significantly from *C. elegans* anatomy (Han et al., 2016). Significantly, therapeutic exploitation of the neuropeptidergic system for nematode parasite control is dependent on a basic understanding of signalling peptide biology across the phylum. Consequently, irrespective of the degree of neuronal gene conservation, biological data to support drug target value should be derived from target parasitic nematodes.

## Figures and Tables

**Table 1:** FMRFamide-like peptide encoding gene-11 (*flp*-11) and FMRFamide-like peptide encoding gene-32 (*flp*-32) complement in nematodes. Black shading indicates the presence of a gene.

Servers/databases employed in *flp*-11 and -32 BLAST were as follows: **Server:** NCBI (http://blast.ncbi.nlm.nih.gov/Blast.cgi), **Database(s):** expressed sequence tags (EST), nucleotide collection (NR/NT), genomic survey sequences (GSS), whole-genome shotgun contigs (WGS), high throughput genomic sequences (HTGS), transcriptome shotgun assembly (TSA), **Species:** Nematoda (taxid: 6231); **Server:** Broad Institute Filarial worms (http://www. broadinstitute.org/annotation/genome/filarialworms/Blast.html), **Database(s):** filarial worm transcripts, filarial worm genomic sequences, **Species:***B. malayi, W. bancrofti*, *L. loa*, *O. volvulus*; **Server:** 959 Nematode Genomes (http://xyala.cap.ed.ac.uk/downloads/959nematodegenomes/blast/blast.php), **Databases:** all available, **Species:***D. immitis, O. ochengi, A. suum*, *O. tipulae, B. xylophilus*, *H. polygyrus*, *Heligmosomoides aeoronymphium*, *Litomosoides sigmondontis*, *M. floridensis*; **Server:** WormBase (https://www.wormbase.org/tools/blast_blat), **Version:** WS238-WS247, **Database(s)**: all available, **Species:***T. spiralis*, *T. suis, B. malayi, D immitis, L. loa, O. volvulus, A. suum*, *P. pacificus, P. expectatus, H. contortus, N. americanus, A. ceylanicum, H. bacteriophora, B. xylophilus*, *P. redivivus*, *S. ratti, M. incognita, M. hapla*; **Server:** INRA (http://meloidogyne.toulouse.inra.fr/blast/blast.html), **Database(s)**: Genomic Scaffolds/Contigs, predicted proteins, unplaced reads, **Species:***M. incognita*; **Server:** Welcome Trust Sanger Institute (http://www.sanger.ac.uk/resources/software/blast/),**Database(s):** Worm and Parasitic Helminths; **Species:***T. muris, B. malayi, O. volvulus, A. suum, H. contortus, T. circumcincta, N. brasiliensis*, *S. ratti*, *G. pallida.* BLAST searches were executed on every species available on every database regardless of duplication. *C. elegans* is representative of all *Caenorhabditis* species available on WormBase database.

## Appendix A Supplementary data

Supplementary Fig. S1 Alignment of translated open-reading frame (ORF) amino acid sequences. (A) ORF alignment of FLP-11-like peptide encoding genes in nematode species of interest, showing the three conserved FLP-11 peptides, AMRNALVRFG (highlighted in yellow), A(S/G/A)(G/S)(G/S/M/T)MRNALVRFG (highlighted in pink), and N(G/N)(A/P)PQPFVRFG (highlighted in blue), flanked by conserved di- and mono-basic cleavage sites (highlighted in green). Note: *Caenorhabditis elegans* (*Ce*)*-flp-11* splice variant isoforms (b) and (c) do not encode functional copies of NGAPQPFVRFG (highlighted in blue), *Panagrellus redivivus* (*Pr*)-flp*-11* splice variant isoform (b) encodes only a single copy of NGAPQPFVRFG (highlighted in blue), and *Globodera pallida* (*Gp*)*-flp-11* does not encode a copy of AMRNALVRFG. *Hc-flp-11*, *Haemonchus contortus flp-11*; *Tc-flp-11*, *Teladorsagia circumcincta flp-11*. (B) ORF alignment of FLP-32-like peptide encoding genes in nematode species of interest, showing the single conserved FLP-32 peptide, AMRN(S/A)LVRFG (highlighted in yellow), flanked by di-basic cleavage sites (highlighted in green). *Ce-flp-32, C. elegans flp-32; Hc-flp-32, H, contortus flp-32; Tc-flp-32, T. circumcincta flp-32; Gp-flp-32, G. pallida flp-32*.

Supplementary Table S1 Primer sequences used in the study.
