# Supplementary material for: Unraveling flp-11/flp-32 dichotomy in nematodes
Source: Int J Parasitol. 2016 Oct;46(11):723–36. doi: 10.1016/j.ijpara.2016.05.010 (PMC5038847; doi:10.1016/j.ijpara.2016.05.010)
Supplement: Supplementary Fig. S1 — Alignment of translated open-reading frame (ORF) amino acid sequences. (A) ORF alignment of FLP-11-like peptide encoding genes in nematode species of interest, showing the three conserved FLP-11 peptides, AMRNALVRFG (highlighted in yellow), A(S/G/A)(G/S)(G/S/M/T)MRNALVRFG (highlighted in pink), and N(G/N)(A/P)PQPFVRFG (highlighted in blue), flanked by conserved di- and mono-basic cleavage sites (highlighted in green). Note: Caenorhabditis elegans (Ce)-flp-11 splice variant isoforms (b) and (c) do not encode functional copies of NGAPQPFVRFG (highlighted in blue), Panagrellus redivivus (Pr)-flp-11 splice variant isoform (b) encodes only a single copy of NGAPQPFVRFG (highlighted in blue), and Globodera pallida (Gp)-flp-11 does not encode a copy of AMRNALVRFG. Hc-flp-11, Haemonchus contortus flp-11; Tc-flp-11, Teladorsagia circumcincta flp-11. (B) ORF alignment of FLP-32-like peptide encoding genes in nematode species of interest, showing the single conserved FLP-32 peptide, AMRN(S/A)LVRFG (highlighted in yellow), flanked by di-basic cleavage sites (highlighted in green). Ce-flp-32, C. elegans flp-32; Hc-flp-32, H, contortus flp-32; Tc-flp-32, T. circumcincta flp-32; Gp-flp-32, G. pallida flp-32. [file mmc1.docx]

**A**

1 76

*Ce-flp-11*(a) (1) -----------------------------MTQFSALALLLIVFVAASFAQSYDDVSAEKRAMRNALVRFGRASGGM

*Ce-flp-11*(b) (1) -----------------------------MTQFSALALLLIVFVAASFAQSYDDVSAEKRAMRNALVRFGRASGGM

*Ce-flp-11*(c) (1) -----------------------------MTQFSALALLLIVFVAASFAQSYDDVSAEKRAMRNALVRFGRASGGM

*Hc-flp-11* (1) ------------------------------MTSSTTIKFCIIAVVIASLRAQDSFALEKRAMRNALVRFGRAGGSM

*Tc-flp-11* (1) ------------------------------MPSSTSMKLCLVAMLVASIWAQDDSALEKRAMRNALVRFGRAGGSM

*Pr-flp*-*11*(a) (1) -----MDLDVATHLFAARNIVSVIKMQASSVLLFGLALAFCVIVSNAQFDEDYAAPTEKRAMRNALVRFGRAAGM-

*Pr-flp-11*(b) (1) ------------------------------MDLDVATHLFAARNIVSVIKMQASSVLLFGLALAFCVIVSNAQFDE

*Gp-flp-11* (1) MIISTNAASITDVEDGQQQQQQQQQQPFALFPVASMPAEEFGGDGGGFIGSLPQMAKRRQQLLNALLMTRRLGRSP

77 151

*Ce-flp-11*(a) (48) -----------RNALVRFGKRSPLDEEDFAPESPLQGKRNGAPQPFVRFGRS-GQLDHMHDLLSTLQKLKFANNK

*Ce-flp-11*(b) (48) -----------RNALVRFGKRSPLDEEDFAPESPLQGKRNGAPQPFEAQVRQ-QQVMTEDDRLLLEQLLRRIHH-

*Ce-flp-11*(c) (48) -----------RNALVRFGKRSPLDEEDFAPESPLQGKRNGAPQPFGKLS-------------------------

*Hc-flp-11* (47) -----------RNALVRFGKRY-LATDDDYATAAAQGKRNGAPQPFVRFGRS-GHLDHIHDILSTLQKLQLANYH

*Tc-flp-11* (47) -----------RNALVRFGKRS-STADDDYAAAVAQDKRNGAPQPFVRFGRS-GHLDHIHDILSTLQKLQMANYY

*Pr-flp-11*(a) (71) -----------RNALVRFGKRS-ADEIAVMPDYGNEAKRNGAPQPFVRFGRSAGRIDHMHDILSTLQKIEMANGQ

*Pr-flp-11*(b) (47) -----------DYAAPTFGKRS-ADEIAVMPDYGNEAKRNGAPQPFVRFGRSAGRIDHMHDILSTLQKIEMANGQ

*Gp-flp-11* (77) RASVRLPRSTMRNALVRFGKRA-------LLPMMALDDKRNPPQPFVRFGRSAAANAVENGATDPMFFAVS----

**B**

1 75

*Ce-flp-32* (1) --------MLSFVQTLILALLCSIVFVEAMPSMRPAKKAMRNSLVRFGKRADPVGTDDVFLGESYGSADPYEYVP

*Hc-flp-32* (1) --------------FVLTLFISMLIVEAALPRMRHTKRAMRNSLVRFGKRADLSDVVLLEEPSGIAESDLFYSGV

*Tc-flp-32* (1) ---------MIGRSFVLTLFISMLIVEAALPRMRHTKRAMRNSLVRFGKRADLSDVVLLEEPSGIADSDLFYSGV

*Pr-flp-32* (1) ----MNATRYLLRLIFAAVMISLIASEALSTRVRPDKKAMRNSLVRFGKRAEVAAYAPLDDDAVQDSSIVDTPWA

*Gp-flp-32* (1) MSKLCRFSRFFYAVLLLLTLCSILMADAAVWRMRTDKKAMRNALVRFGKRNAYRSSGEAFVGAAGFGDSGAHLLR

76 107

*Ce-flp*-*32*  (68) ERMSNRGPSSVLLY------------------

*Hc-flp-32* (62) AQPR----------------------------

*Tc-flp-32* (67) AQPRNQLRTLYN--------------------

*Pr-flp-32* (72) YNGNRFIYPRIQDLIQ----------------

*Gp-flp-32* (76) DIGMDGRQTQWAAFGDGGAPRPIKRLLLWPEQ

**Supplementary Fig. S1.**
