# Supplementary material for: Unraveling flp-11/flp-32 dichotomy in nematodes
Source: Int J Parasitol. 2016 Oct;46(11):723–36. doi: 10.1016/j.ijpara.2016.05.010 (PMC5038847; doi:10.1016/j.ijpara.2016.05.010)
Supplement: Supplementary Table S1 — Primer sequences used in the study. [file mmc2.docx]

**Supplementary Table S1.** Primer sequences used in the study.

| **Gene name**  **(primer use)** | **Oligonucleotide sequence (S: 5’-3’; A: 3’-5’)** | **Amplicon size (bp)** |
| --- | --- | --- |
| ***Ce-flp-11* (ISH^a^)** | S: CAATTCTCTGCATTGGCACT  A: TGGAGCAAAGTCTTCCTCGT | 195 |
| ***Ce-flp-32* (ISH)** | S: CCCTTATCCTCGCTCTCCTC  a: CGCTCCGGAACATATTCATAC | 187 |
| ***Gp-flp-11* (GSP ORF^b^)** | S: ATGATCATCTCCACCAATGCC  a: CCATTTTCGACGGCATTC | 389 |
| ***Gp-flp-11* (ISH)** | S: CAGCAGCAACAACAACAACC  A: CATTGTCGACCGAGGTAGG | 201 |
| ***Pr-flp-11* (DGP)** | S: GCIATG(A/C)GIAA(T/C)GC(G/A/T/C)(C/T)TIG  (G/A/T/C)(A/C)G  A: AC(G/A)AAIGG(C/T)TGIGG(G/A/T/C)CC(A/G)TT | 148 |
| ***Pr-flp-11* (GSP ORF)** | S: ATGGACTTGGATGTTGCGACC  A: TGgccattggccatctcaatct | 398 |
| ***Pr-flp-11* (ISH)** | S**:** AAACGCTCTGCTGACGAAATCGC  a: CGCAAGCGATTAAAAGGAACA | 223 |
| ***Tc-flp-11* (SL1/DGP)** | S**:** GGTTTAATTACCCAAGTTTGAG  a: AC(G/A)AAIGG(C/T)TGIGG(G/A/T/C)CC(A/G)TT | >252 |
| ***Tc-flp-11* (GSP ORF)** | S**:** ATGCCATCGTCTACGTCGATG  a: TCAGTAATAGTTGGCCATCTG | 327 |
| ***Tc-flp-11* (ISH)** | S**:** TTTGTGCTGACGCTGTTCAT  a: ttcgaggttgtgctactcca | 196 |
| ***Tc-flp-32* (GSP ORF)** | S**:** ATGATTGGTCGCTCCTTTGT  A: TTGCGGACTTTATATAACTGA | 237 |
| ***Hc-flp-11* (GSP ORF)** | S**:** ATGACATCGTCCACTACGATAAA  a: TCATGCCATGACAGATGAAGG | 587 |
| ***Hc-flp-11* (ISH)** | S**:** CAAAAGCTGCAACTGGCTAA  a: gacagagacctgccacaaaa | 197 |
| ***Hc-flp-32* (GSP ORF)** | S**:** ATGATTGGTCGCTCCTTTGT  a: TTGCGGACTTTATATAACTGA | 237 |

^a^ISH denotes primers used to generate in situ hybridisation templates and probes.

^b^GSP ORF denotes gene-specific primers used for open reading frame confirmation.

SL-1, nematode splice leader primer; DGP, degenerate primer; *Ce*, *Caenorhabditis elegans*; *Gp,* *Globodera pallida*; *Pr*, *Panagrellus redivivus*; *Tc,* *Teladorsagia circumcincta*; *Hc*, *Haemonchus contortus*.
